# Machine-Learning-Assisted Carbon Dots: From Algorithms to Applications and Beyond

**DOI:** 10.3390/molecules31101696

**Published:** 2026-05-17

**Authors:** Fengjiao Jia, Hengkai Wang, Deyu Shen, Dandan Sang, Zhanfeng Zhang, Hang Li, Santosh Kumar, Qinglin Wang

**Affiliations:** 1 School of Physics Science and Information Technology, Liaocheng University, Liaocheng 252000, China; 2023403960@stu.lcu.edu.cn (F.J.); 2021403207@stu.lcu.edu.cn (H.W.); a15163416966@126.com (D.S.); 2 Shandong Jinhengli Mechanical Manufacturing Co., Ltd., Taian 271200, China; 13345290998@163.com (Z.Z.); 18653802385@163.com (H.L.); 3 Centre of Excellence for Nanotechnology, Department of Electronics and Communication Engineering, Koneru Lakshmaiah Education Foundation, Vaddeswaram 522302, Andhra Pradesh, India; santosh@kluniversity.in

**Keywords:** carbon dots, machine learning, extreme gradient boosting, quantum yield, photoluminescence, carbon dots sensor, antibiotic

## Abstract

Carbon dots (CDs) have emerged as frontier materials in multidisciplinary research owing to their unique optical properties and physicochemical characteristics. However, issues such as the reliance on trial-and-error experimentation for synthetic preparation and the difficulty in systematically revealing structure–activity relationships persist. In recent years, machine learning (ML) has provided a new paradigm for CD research through its powerful predictive and decision-making capabilities. This review first introduces the fundamental workflow of ML and the operational principles of several representative ML algorithms. It then summarizes the ML applications in CDs, including ML-optimized CD synthesis, ML-assisted detection in CD sensors, ML-based performance prediction, and ML-driven mechanism studies. Finally, the review outlines the future prospects for applications in this field, aiming to further advance the development of nanomaterials science.

## 1. Introduction

Carbon dots (CDs), an emerging class of zero-dimensional carbon-based nanomaterials, exhibit broad application potential across various fields owing to their outstanding biocompatibility, tunable optical properties, low toxicity, high conductivity, and facile synthesis [1,2,3]. In terms of optical properties, CDs have the characteristics of high photostability, tunable luminescence and broad excitation spectrum, but their photoluminescence mechanism is still controversial and may be related to factors such as conjugated structure, surface state or molecular state [4,5]. In the field of biomedicine, CDs are widely used in bioimaging (such as in vitro cell labeling and in vivo tumor imaging), biosensing (such as the detection of metal ions, small molecules, and biopolymers), and treatment (such as photothermal therapy and drug delivery) [6,7,8]. Additionally, they exhibit excellent catalytic activity and electron mobility in electrocatalysis and energy devices [9]. Typically, at room temperature, CDs can be synthesized via top-down and bottom-up methods. The latter are more widely used because of their superior versatility and accessibility. Among these, environmentally benign methods, such as hydrothermal and microwave-assisted synthesis, are attractive because they use readily available, low-cost raw materials. At low temperatures, carbon dots with high quantum yields and excellent performance can be synthesized for use as fluorescent probes [10,11]. Biomass-derived CDs are regarded as an ideal alternative material for addressing issues such as the high toxicity and poor environmental compatibility of conventional semiconductor quantum dots [12,13]. However, CDs possess a wide variety of surface functional groups (such as amino, hydroxyl, and aldehyde groups), and their differing sizes, shapes, and anchoring methods result in complex structures, often making their synthesis difficult to control [14]. At the same time, optimizing the synthesis conditions of CDs often involves a complex trial-and-error process with limited predictability, leading to extended timelines and reduced synthesis efficiency [15]. Their practical applications still face challenges, including the absence of clear structure–activity relationships, structural inconsistencies, fluctuations in quantum yield, and potential cytotoxicity caused by degradation under light exposure [16,17,18].

As a core discipline of artificial intelligence, machine learning (ML) utilizes algorithmic models to enable computers to autonomously learn patterns and rules from data, thereby achieving prediction, classification, and optimization of complex systems. This approach has significant value in modern scientific research, particularly in contexts where data volume surpasses human analytical capacity or where underlying relationships are inherently complex. Therefore, it has great application potential in research fields such as biomedicine (analyzing genome and clinical data, studying disease mechanisms) [19], materials science (optimizing material properties, designing and discovering various nanomaterials) [20,21,22,23], fluid mechanics [24,25], industrial manufacturing (intelligent upgrades of predictive maintenance, quality control, and supply chain management) [26], and chemical reaction detection [27]. For instance, Zhang et al. [28] achieved the efficient substitution of the scarce element cobalt (Co) by using ML to interpret the influence of elements on alloy properties. Wen et al. [29] successfully screened refractory high-entropy alloys with both excellent strength and room-temperature ductility using a support vector regression model, nondominated sorting genetic algorithm, and K-means clustering algorithm (K-MCA), fully demonstrating the effectiveness of ML-assisted material design. In extreme environments, ML also plays a vital role. Under low-temperature conditions, ML not only predicts the key properties of sealing materials and deep-freeze damage factors in concrete but also identifies *Cenchrus fungigraminus* and elucidates the drying process of sludge [30,31,32,33]. Under high-temperature and high-pressure conditions, ML has enabled the prediction of material properties across diverse domains, including mechanics (elastic constants, phonon frequencies), thermodynamics (thermal conductivity, free energy), and electricity (superconductivity, electrical conductivity) [34,35,36,37,38].

**Figure 1:**
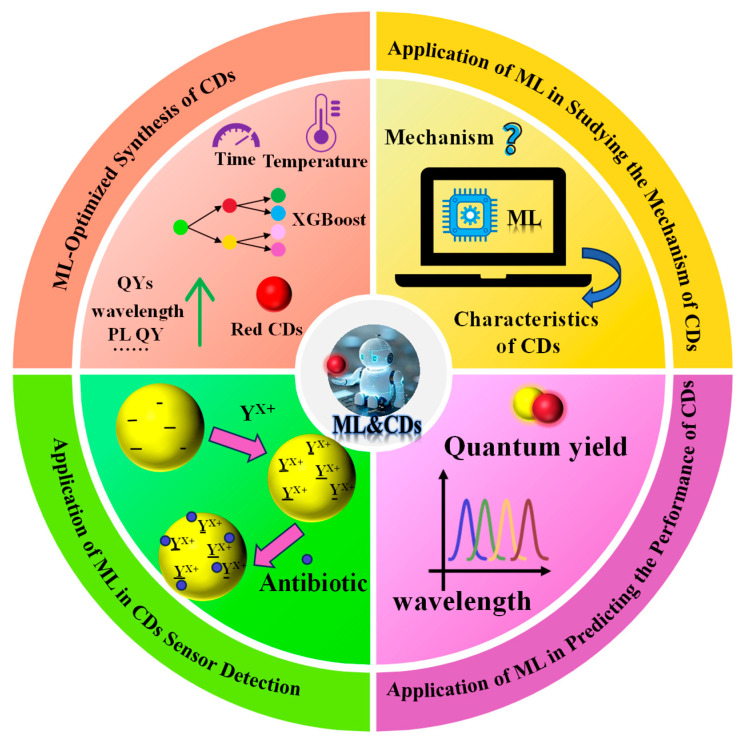
This review systematically outlines the application of ML in CDs research: ML optimization of CDs synthesis, ML-assisted detection using CDs sensors, ML for performance prediction, and ML in studying mechanisms.

Currently, ML is applied not only across diverse fields such as biomedicine, materials science, and chemical reaction detection, but it has also demonstrated distinct advantages and potential in CD research. For instance, Han et al. [39] employed the extreme gradient boosting (XGBoost) algorithm to construct human-interpretable decision trees, effectively guiding the synthesis of CDs with a QY of 39.3%. Pandit et al. [40] reported a CD-based sensor array that, when combined with ML techniques, can distinguish between eight proteins with 100% accuracy. Chen et al. [41] developed an ML strategy that successfully predicted the yield of CDs generated during the biochar production process. This review first outlines the workflow of ML and provides a detailed analysis of how typical ML algorithms operate. Subsequently, it comprehensively summarizes the applications of ML in optimizing CD synthesis, assisting CD sensors in detection, predicting performance, and investigating mechanisms (Figure 1). Finally, it discusses the challenges faced in applying ML in the CD field and explores future development prospects.

## 2. The Workflow of ML

ML is a system that learns from data through training, with its core function being the modelling of data and the optimization of model parameters via algorithms. A typical ML model follows a systematic workflow, which is crucial for studying its applications and principles across various domains. The general workflow of ML primarily encompasses data collection and preprocessing, model selection and training, and model evaluation and prediction.

### 2.1. Data Collection and Preprocessing

First, the ML process requires the collection and preparation of data as inputs to the algorithm. Generally, data sources include previously published papers, sample sets generated through experiments or simulations, and databases [42,43]. A dataset can be divided into a training set (used for model learning), a validation set (used for parameter tuning and model selection), and a test set (used to evaluate model performance) [44]. To reduce the randomness caused by data partitioning, cross-validation can be performed on the dataset. This involves dividing the dataset into several subsets, then taking turns using one subset as the test set and the rest as the training set, and calculating the average error after multiple iterations. Data quality plays a crucial role. Due to the potential presence of missing values, outliers, and redundant data in the raw data, preprocessing of the collected data is necessary. For example, in their study on the relationship between the physicochemical characteristics and antimicrobial activity of CDs, Bian et al. [45] encountered over 50% missing values for both hydrodynamic size and chemical composition. Consequently, they excluded features with higher proportions of missing values and selected those relevant to the learning process. To enhance data processing speed, enable data visualization, and preserve the most critical features of high-dimensional data, dimensionality reduction is also required. For instance, Talukder et al. [46] employed principal component analysis (PCA) for dimensionality reduction to enhance computational efficiency in intrusion detection for wireless sensor networks. At the same time, data standardization must be performed to scale feature data with different units and ranges to a common scale, ensuring the stability and efficiency of model training. If categorical variables are present in the data, the model cannot directly process this information. Feature encoding is required to convert categorical variables into numerical variables. Common encoding methods include one-hot encoding and label encoding. The former converts each category into a binary variable, which is suitable for unordered categories. The latter directly maps the category to an integer, which is suitable for ordered categories.

### 2.2. Model Selection and Training

The suitability of an ML model depends on the nature of the problem (classification, regression, clustering, reinforcement learning, and anomaly detection) and the characteristics of the data [47]. ML models can generally be categorized into two types: supervised and unsupervised. Supervised learning trains models using labeled data. In supervised learning, each training example requires both an input object and a corresponding output object, enabling the model to make predictions on new data. It can be categorized into two main types based on the nature of the target variable being predicted: classification and regression. Classification aims to predict a discrete, finite category label, whereas regression predicts a continuous numerical value. Common algorithms include linear regression, linear discriminant analysis (LDA), support vector machines (SVM), random forests (RF), gradient-boosted decision trees (GBDT), and artificial neural networks (ANN) [48,49,50]. For example, Mandal et al. [50] utilized the fluorescence response of carbon nanoparticles (CNPs) to predict heavy metal ions. This prediction was performed by mapping input variables to output variables, thus employing supervised learning. Unlike supervised learning, unsupervised learning is a statistical approach that uncovers underlying structures or distributions from unlabeled data. It relies on the inherent characteristics of the data itself for clustering, dimensionality reduction, and other operations. Cluster analysis groups data points into clusters, where data points within the same cluster are similar to each other. Dimensionality reduction is required to facilitate data analysis and visualization. This process maps high-dimensional data to a low-dimensional space while preserving as much original information as possible. Common algorithms include mean-based clustering, PCA, and hierarchical cluster analysis (HCA). Feature extraction using K-MCA generates K features from the R, G, and B channels, thereby improving the output accuracy of ML models [51]. Applying HCA to partition samples into distinct subsets facilitates the extraction of interpretable patterns, thereby enhancing the accuracy of predictive models [52]. Performing PCA on data reduces dimensionality [53], eliminating the influence of raw data and enabling regression analysis. However, ML sometimes relies excessively on manual feature engineering, making it challenging to handle complex nonlinear relationships. Deep learning, as a subset of ML, can automatically extract features through multi-layer neural networks (such as convolutional neural networks) and is primarily suited for unstructured data.

### 2.3. Model Evaluation and Prediction

By comprehensively evaluating the performance metrics of a model, one can more systematically assess its predictive accuracy and generalization capability. Generally, for regression problems, three performance metrics are commonly used to evaluate the performance of ML models: the coefficient of determination (R^2^), mean squared error (MSE), and Pearson correlation coefficient (r) [39]. Common metrics include the mean absolute error (MAE), root mean square error (RMSE), and mean absolute percentage error (MAPE) [43]. In classification problems, model performance is often evaluated using metrics such as accuracy, precision, recall, F1 score, and the area under the receiver operating characteristic curve (AUC-ROC curve). To pursue optimal model generalization, cross-validation and hyperparameter optimization are sometimes employed to prevent overfitting or underfitting.

Precision is the proportion of samples with a true value of 1 among all samples predicted as 1. The equation is

(1) $$P=\frac{TP}{TP+FP}.$$

Recall is the proportion of samples predicted as 1 among all samples with a true value of 1. The equation is

(2) $$r=\frac{TP}{TP+FN}.$$

The F1 score is a metric used to measure the accuracy of binary classification models and simultaneously accounts for both precision and recall. The equation is

(3) $$F1=\frac{2Pr}{P+r}.$$

TP, FP, and FN refer to true positives, false positives, and false negatives, respectively. For example, Liu et al. [54] employed precision, recall, and the F1 score as evaluation metrics to validate the performance of the You Only Look Once (YOLO) v3 model, ultimately calculating precision at 92.5%, recall at 100.0%, and the F1 score at 96.1%. The model demonstrated high accuracy and excellent performance. Similarly, the AUC-ROC curve serves as a crucial tool for evaluating the performance of binary classification models. The ROC curve plots the relationship between the true positive rate and the false positive rate at different classification thresholds. The AUC represents the classifier’s ability to distinguish between positive and negative samples. Specific methods for measuring performance within the model are discussed in the following sections.

### 2.4. Common ML Algorithms

SVMs are powerful and flexible supervised learning algorithms primarily used for classification and regression tasks. The core idea of this algorithm is to find a decision boundary (hyperplane) with the maximum margin. However, this algorithm can only handle linearly separable data. Kernel functions have been introduced to address linearly inseparable data. For example, Liu et al. [55] employed kernel functions to address the nonlinear problem of identifying multiple lithofacies types. Through a nonlinear mapping, linearly inseparable data in the original low-dimensional space are transformed into a high-dimensional feature space, where the data become linearly separable. This approach flexibly resolves complex nonlinear problems. For example, Liu et al. [42] found that the SVM model performed optimally (test set R^2^ = 0.927) when using ML to investigate the properties of cement-based materials, enabling the capture of nonlinear trends in the data without complex optimization.

In unsupervised machine learning algorithms, the K-MCA is a fundamental, important, and widely applied learning algorithm. The general procedure involves randomly selecting K data points from a dataset containing N data points as initial cluster centers. The distance between each data point and all K cluster centers is calculated, and each data point is assigned to the nearest cluster center, forming K temporary clusters. Subsequently, the cluster center for each cluster is recalculated, which is the average of all data points within that cluster. The iterative process continues until convergence is reached, at which point the cluster centers no longer undergo significant changes. The quality of clustering can be assessed using the silhouette coefficient; the closer this coefficient is to 1, the better the clustering results. However, this algorithm requires K to be specified in advance; however, it is difficult to guarantee the number of meaningful values that exist in the data during actual processing. Because cluster-centered calculations are based on the average of all points within a cluster, they are highly susceptible to the influence of outliers [56]. Unlike K-MCA, HCA does not require a predefined K value or a specified initial center. It generates a dendrogram by splitting or merging samples layer by layer through top-down splitting and bottom-up aggregation, providing a visual representation of the relationships and classifications among samples [52]. For small sample sizes where exploratory data grouping is required, HCA yields more robust clustering results than K-MCA.

Ensemble learning is a highly significant learning method in ML and has been widely applied in industrial and medical fields in recent years. Decision tree ensemble learning refers to the use of multiple decision tree models of the same type, employing ensemble learning methods to enhance model performance and stability. Common algorithms include RF and GBDT. RF is a method that constructs multiple decision trees and averages their results to reduce overfitting. In contrast, GBDT is an ensemble approach that progressively builds decision trees to correct errors made by the preceding tree.

ML platforms and tools are indispensable for the efficient execution of modern machine learning and deep learning tasks. Representative examples based on the Python ecosystem include scikit-learn and TensorFlow. Scikit-learn is recognized as a premier library for traditional ML, distinguished by its comprehensive documentation and extensive suite of data processing utilities. It is commonly employed for data preprocessing, classification, regression, and dimensionality reduction, and supports the entire workflow—from feature engineering and model training to hyperparameter optimization and cross-validation—on conventional computing servers. For deep learning applications, TensorFlow serves as a primary framework [57]. At its core, TensorFlow represents computations using tensors and organizes operations within a dataflow graph architecture to facilitate model training. Notably, TensorFlow provides developers with the flexibility to design and train custom algorithms, making it particularly suitable for research and industrial-scale projects that demand precise control over model architecture and training procedures.

## 3. Application of ML in CDs

### 3.1. ML-Optimized Synthesis of CDs

CDs have garnered significant attention owing to their outstanding optical properties, straightforward synthesis routes, and diverse synthetic approaches [58]. However, the preparation of high-performance CDs not only depends on multiple factors, such as precursors, temperature, and reaction time [1], but even minor variations in the synthesis parameters can influence the properties of CDs [59]. Furthermore, screening CDs with unique optical properties typically relies on extensive trial-and-error experiments, a process that is both time-consuming and costly. Consequently, introducing ML as an auxiliary tool is regarded as an effective solution. By learning from and analyzing existing experimental data, ML methods can build predictive models, optimize parameters, and identify the optimal synthesis conditions, thereby enhancing the performance of CDs. At the same time, this approach can improve the efficiency of synthesizing specific CDs.

#### 3.1.1. Single Performance of ML-Optimized CDs

**Figure 2:**
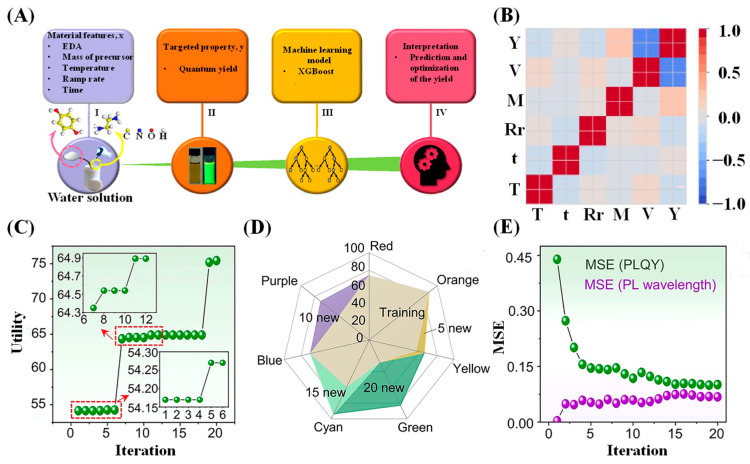
(**A**) Design framework for synthesizing CDs with high QY based on ML and hydrothermal experiments. (**B**) A heatmap of the Pearson correlation coefficients between selected characteristics of hydrothermally grown CDs. T: Temperature; t: time; Rr: Ramp rate; M: Mass; V: Volume; Y: Yield. Reprinted with permission from Ref. [39]. Copyright 2020, American Chemical Society. (**C**) Unified objective utility of MOO and design iterations. (**D**) Exploration of color under new synthetic experimental conditions. (**E**) MSE between predicted and actual target properties. Reprinted with permission from Ref. [64]. Copyright 2024, Nature Communications.

CDs are widely used in the field of fluorescence sensing due to their excellent optical properties, including tunable emission wavelengths, high fluorescence quantum yield (QY), and good stability. Among these, QY is a key indicator of fluorescence emission efficiency, and understanding the relationships among synthetic parameters is crucial for optimizing QY. Han et al. [39] established a regression ML model for hydrothermally synthesized CDs to guide the synthesis of CDs with high QYs. Their design framework is illustrated in Figure 2A. They selected five key parameters (ethylene diamine (EDA) volume, precursor mass, reaction temperature, heating rate, and reaction time) as input features, with QY as the output target, thereby constructing the dataset. As shown in the correlation heatmap in Figure 2B, the selected features exhibit low correlation and high independence, confirming their validity. They subsequently evaluated the XGBoost regressor, multilayer perceptron (MLP), and SVM using three performance metrics: R^2^, MSE, and r. Based on the evaluation results, they selected the XGBoost model. Furthermore, using feature importance analysis, they determined that the key parameters in order of importance were the EDA volume, precursor mass, and reaction temperature. Finally, they screened the optimal synthesis conditions, conducted experimental verification, and obtained CDs with a high QY of 39.3%.

ML can also guide the synthesis of CDs with tunable third-order nonlinear optical susceptibility $\chi^{\left(3\right)}$ and switching behavior. Nonlinear optics has important applications in laser technology, optical communications, and biomedical imaging [60,61,62]. Wang et al. [63] were the first to apply ML to guide the tunable third-order nonlinear optical susceptibility $\chi^{\left(3\right)}$ and switching behavior. First, they select input features based on the synthesis conditions of CDs. Then, they construct experimental datasets under different experimental conditions and perform ML training. Furthermore, they employed four regression models (RF, XGBoost, SVM, and GBDT) and evaluated their performance, concluding that the GBDT model demonstrated optimal performance. Subsequently, they utilized the GBDT model for training and achieved significant progress in the data. Furthermore, they derived feature importance, revealing that reaction time accounted for 87% and reaction temperature for 8.9%. This indicates that the descriptor importance determining $\chi^{\left(3\right)}$ is, in order of significance, reaction time, temperature, and so forth. Therefore, they optimized and tuned the features, successfully synthesizing CDs with $\chi^{\left(3\right)}$ tunable from 0 to 1.79 × 10^−8^ esu, exhibiting excellent nonlinear optical properties and switching behavior.

#### 3.1.2. Multi-Objective Optimization of ML-Based CDs

In ML-guided CD synthesis applications, most approaches optimize a single property by identifying the relationship between parameters and performance. In practical applications, it is often necessary to obtain CDs with multiple outstanding properties. Guo et al. [64] proposed an ML-guided multi-objective optimization strategy to indirectly guide CD synthesis by optimizing multiple properties (photoluminescence (PL) wavelength and PLQY). This strategy comprises four components: database construction, multi-objective optimization formulation, multi-objective optimization recommendation, and experimental validation. They employed an XGBoost ML model to construct a PL wavelength and PLQY prediction model based on eight synthetic parameters (temperature, time, catalyst, etc.). Notably, they adopted an iterative experimental optimization strategy, achieving full-color CDs with PLQY exceeding 60% across all colors after 20 iterations (40 experiments) (Figure 2C,D). The predictive accuracy of the ML model also improved with the number of iterations. As the number of iterations increased, the mean square error (MSE) for PLQY decreased from 0.45 to 0.1, whereas the MSE for PL wavelength remained consistently below 0.1, fully demonstrating the reliability of this approach (Figure 2E).

#### 3.1.3. ML-Guided Synthesis of Specific CDs

Red CDs exhibit high resistance to photo-bleaching and are biodegradable; however, their synthesis efficiency is low. To enhance the synthesis efficiency of red CDs, Luo et al. [58] first reported the use of ML to guide their synthesis. First, they collected synthetic data, then preprocessed it, which included labeling the data and splitting the dataset. Notably, this process employs multi-step feature engineering (XGBoost, one-hot encoding, and PCA). The XGBoost model was employed to extract data by combining input features across multiple dimensions. Categorical features were converted into numerical values using one-hot encoding, transforming each category into a binary vector. This process generated redundant information, prompting the use of PCA for dimensionality reduction by maximizing sample dispersion. Logistic regression was then employed to predict the synthesis conditions for red CDs. Ultimately, the constructed ML model achieved an AUC of 0.94 and an F1 score of 0.94 in 10-fold cross-validation, identifying 93% of red CDs and 95% of non-red CDs, thereby enhancing the efficiency of synthesizing red CDs.

### 3.2. Application of ML in CD Sensor Detection

ML not only optimizes CD synthesis but also assists in distinguishing ions from organic compounds, thereby enhancing detection accuracy and speed. CDs function as fluorescent sensors by utilizing their outstanding fluorescent properties, such as high quantum yield and excellent photostability, to achieve highly sensitive and selective detection of target substances (ions and molecules) through changes in fluorescence signals (intensity and wavelength). Compared to traditional fluorescent probes, CD-based sensors offer advantages such as low toxicity, excellent biocompatibility, and low preparation costs. In the following section, we systematically elaborate on the role of ML in CD sensors.

#### 3.2.1. Applications in Ion Detection

With the advancement of scientific inquiry, significant progress has been achieved in the application of CDs for metal ion detection. In recent years, extensive research efforts have been directed toward the development of CD-based fluorescent sensors for ion detection, as exemplified in Table 1.

By combining ML with fluorescence visualization, Zhang et al. [53] achieved the sequential quantitative detection of Al^3+^ and F^−^ in aqueous solutions. They employed synthetic sulfur-functionalized CDs as fluorescent probes and observed enhanced fluorescence intensity upon adding Al^3+^ at varying concentrations to the CDs solution, achieving a detection limit of 4.2 nmol/L. Fluorescence quenching occurred upon the addition of F^−^. This phenomenon arises when quenchers interact with fluorescent molecules through processes such as charge transfer, electron transfer, or coordination bonding, leading to signal attenuation. The resulting detection limit was 47.6 nmol/L, demonstrating an “off-on-off” detection mode. They employed K-MCA, evaluating clustering tendency via Hopkins statistics: Hopkins statistics for Al^3+^ was 0.96, and for F^−^ it was 0.95. They then used PCA to reduce the dimensionality of three-dimensional variables to one-dimensional variables by comparing the magnitude of variance. The heatmap shows that there is a positive correlation with Al^3+^ and a negative correlation with F^−^. Therefore, a linear regression model can be constructed based on the linear relationship between them. By predicting the concentrations in the test dataset, the final R^2^ values obtained were 0.927 for Al^3+^ and 0.987 for F^−^, indicating that this model can be used for predicting and detecting pollutant concentrations.

ML can also assist in the detection of toxic heavy metal ions. Mandal et al. [50] developed an array sensor based on CNPs and integrated it with the predictive capabilities of artificial intelligence. This study achieved the first detection and differentiation of five toxic heavy metal ions (As (III), Cd (II), Hg (II), Cr (VI), and Pb (II)) listed by the World Health Organization and the U.S. Occupational Safety and Health Administration. The researchers analyzed sensor response images using multiclass classification algorithms, representing each pixel with red, green, and blue values. The extracted RGB dataset was then employed for pattern recognition. Testing revealed that deep-learning-based supervised algorithms enhanced the MLP to achieve optimal performance. The sensor array successfully distinguished heavy metal ions in labeled river water from those in sewage, demonstrating the concept of utilizing optical sensor data for accurate analytical predictions. First, the researchers synthesized nine types of CNPs with different surface functionalizations at a concentration of 50 ng μL^−1^ and characterized their structure, morphology, and photophysical properties. Subsequently, they constructed a fluorescent array sensor exhibiting distinct visual responses to toxic heavy metal ions, where interactions between different CNPs and heavy metal ions induced fluorescence changes. During this process, they considered the RGB values at 20 positions within the image and recorded the fluorescence responses of the nine distinct CNPs to each heavy metal ion. RGB values were extracted from digitally captured fluorescence images of each CNP–heavy-metal-ion combination to create a dataset, which was divided into training and testing sets for algorithmic learning and decision-making. Seven multiclass classification algorithms were then applied to analyze the sensor array data. The final results show that the enhanced MLP outperformed other methods, with the model converging after training. Concurrently, the average values for sensitivity, specificity, positive predictive value, and negative predictive value across all category labels were 92.45%, 79.67%, 93.75%, and 95.83%, respectively, demonstrating high accuracy even on a small dataset. Ultimately, the enhanced MLP was identified as optimal, validating the feasibility of the sensor array in real water samples. Notably, the pioneering use of generative adversarial networks (GANs) to augment the dataset enhanced the performance of deep learning algorithms in the automated heavy metal detection platform based on CNPs. The synthesized alizarin red S-based carbon nanoparticle fluorescent array sensor, combined with AI predictive analytics, effectively distinguished toxic heavy metal ions. The enhanced MLP algorithm demonstrated outstanding performance, whereas the sensor array exhibited robustness and practicality in real-world water sample detection. This proves that optical sensor data can be utilized for accurate analyte prediction without direct manual intervention.

Zhang et al. [51] developed an integrated ML approach to meet the demand for real-time, highly sensitive on-site detection of Cr (VI) in groundwater and drinking water. First, they synthesized N-doped blue light carbon dots (N-BCDs) with a QY of approximately 90%. Cr (VI) can be detected within 1 min by utilizing the internal filtering effect (IFE) quenching. Subsequently, they analyzed the fluorescence response of N-BCDs toward Cr (VI) at different concentrations using the Stern–Volmer plot of Cr (VI):

(4) $$\frac{I}{I_{0}}=I+K_{SV}[M].$$

where $I_{0}$ and I are the luminescence emission intensities of the N-BCD analyte suspension, [M] is the molar concentration of the analyte (mM), and $K_{SV}$ is the Stern–Volmer constant. Calculations indicate a detection limit of 0.1574 μgL^−1^ for Cr (VI) concentrations ranging from 0 to 60 μgL^−1^. Furthermore, the fluorescence intensity at 425 nm progressively decreases with increasing Cr (VI) concentration. Through iterative adjustments of the cluster centers, compactness is achieved within each cluster. Subsequently, RGB and K-means feature extraction were applied in combination with ridge, XGBoost, SVR, and linear models to determine the concentrations. The final results demonstrated that the RGB and K-means feature extraction approach, integrated with ML models, achieved a fitting accuracy of 95.2%.

The combination of ML technology with nanoparticle fluorescence sensors enables not only the detection of individual chromium species but also the simultaneous quantification and identification of multiple chromium species. Khozani et al. [65] overcame the limitations of traditional chromium speciation methods by proposing a single-pore fluorescent sensor. This sensor utilizes orange-emissive thioglycolic acid-stabilized CdTe quantum dots (TGA-QDs) and CDs to detect chromium species. ML analysis of fluorescence spectral changes enables the quantitative detection and identification of multiple chromium species. The fluorescence spectrum of the QDs-CDs nanoprobe varies with concentration. Concurrently, adding different chromium species induces distinct spectral shifts, thereby enabling species differentiation based on quenching intensity. Partial least squares (PLS) regression modeling demonstrates strong linearity for Cr^2+^, Cr^3+^, and CrO_4_^2−^ within a specific concentration range. Thus, the QDs-CDs nanoprobe enables the simultaneous measurement of different chromium species. Subsequently, the authors employed an LDA model to distinguish individual chromium species from binary mixtures. The two-dimensional LDA score plot clearly delineated the differences between individual chromium species at varying concentrations. In practical water samples, both ML models were utilized for qualitative and quantitative analysis, thereby extending the application of the sensor to complex environmental samples.

**Table 1:** Examples of ions recently detected by CD sensors.

Detected Ions	Detection Limit	Detection Range	Sensor Materials	References
Cr^6+^ Fe^2+^ Fe^3+^ Mn^2+^ Cu^2+^ Co^2+^ Ni^2+^	0.05 μM	-	QR-CDs EDTA-Tb^3+^	[66]
Cr^6+^ Fe^2+^ Fe^3+^ Hg^2+^	-	1–50 μM	CPC-CDs	[67]
Cd^2+^ Pb^2+^ Hg^2+^	0.15 μM 0.20 μM 0.09 μM	-	AuNCs@NCDs	[68]
Pb^2+^ Fe^3+^	12 nM 16 nM	1–100 μM	VV-CDs	[69]
Hg^2+^	0.06 nM	-	CDs	[23]
Hg^2+^	6.2 nM	-	CQDs	[70]
Fe^3+^	0.135 μM	0.3–3.3 μM	N-CDs	[71]
Fe^3+^	0.039 μM	0–150 μM	CDs	[39]
Fe^3+^	0.91 μM	1–200 μM	CD@Eu-MOF	[72]
Cu^2+^	200 nM	1–100 μM	NS-CDs	[73]
As^3+^	16.8 nM	0–200 nM	CDs-MnO_2_	[74]
Cr^4+^	21.14 nM	0.03–50 μM	S, N-CDs	[75]

#### 3.2.2. Applications in Antibiotic Detection

Antibiotic residues pose serious threats to human health and the natural environment; however, their detection is hampered by high costs and cumbersome procedures. ML algorithms offer the advantages of being responsive, low-cost, simple, and fast, making them suitable for use in antibiotic detection. Relevant applications are shown in Table 2. Xu et al. [49] developed a dual-channel fluorescent sensor array based on two CDs (QR-CDs and CPC-CDs) and utilized ML to detect and distinguish four tetracyclines: Tetracycline (TC), oxytetracycline (OTC), doxycycline (DOX), and minocycline (MTC). Through the IFE, they observed that TCs induced distinct patterns of fluorescence intensity changes in CDs, thereby enabling the detection and differentiation of TCs. IFE refers to the phenomenon wherein the fluorescence intensity diminishes when fluorophores are present at high concentrations or coexist with other light-absorbing substances, owing to the absorption of excitation or emission light by these substances. Subsequently, they employed LDA and SVM to process multidimensional data. At different concentrations, each type of TC did not overlap with others. Ultimately, 100% discrimination of each TC type was achieved.

In array-based antibiotic detection, most arrays only perform detection and differentiation under specific concentrations and sample conditions, making it difficult to detect unknown samples and lacking a unified model. To address these challenges, Xu et al. [76] proposed a novel dual-emission fluorescence/colorimetric sensor array based on IFE, static quenching, and electrostatic interactions. This array utilizes highly fluorescent quantum yield CDs and CdTe quantum dots. Based on the addition of nine different antibiotics (ofloxacin (OFLX), amikacin sulfate (AMK), pefloxacin mesylate (PF), norfloxacin (NFC), kanamycin sulfate (KNM), DOX, metacycline (MTC), TC, and streptomycin (SM)), the fluorescence intensity (FI) and maximum emission wavelength (MEW) undergo changes. These distinct variations enable differentiation among the nine antibiotics. Within the tree-based pipeline optimization technology (TPTO) framework, stepwise prediction strategies were combined with ML to establish classification and concentration models, forming a unified sx model. TPTO is an automated ML tool and a tree-based model pipeline for predicting classification problems. It typically encompasses data cleaning, feature selection, feature processing, feature engineering, model selection, and hyperparameter optimization. By intelligently exploring thousands of potential pipelines, it identifies the optimal pipeline based on the data. Subsequently, the sx model was applied to identify nine antibiotics in deionized water, achieving 95% accuracy. The R^2^ value between the actual and predicted categories reached 0.9888, whereas the R^2^ between the actual and predicted concentrations for unknown samples was 0.9988. Both demonstrated linear relationships and excellent prediction performance. Furthermore, the sx model successfully identified binary and ternary mixtures with 100% accuracy.

To further assist antibiotic detection, Mandal et al. [77] proposed a deep learning recognition method based on nanoparticle array fluorescence imaging, which enabled multicategory antibiotic visualization without spectroscopic instruments. The detection principle relies on distinct fluorescence patterns emitted by all six antibiotics under a 350 nm laser wavelength. The researchers synthesized CNPs and optimized a nine-channel array (including eight metal ion channels) to capture antibiotic-induced fluorescence responses via a digital camera. Specifically, they selected pixel values for the cyan (C), magenta (M), yellow (Y), and key (K) channels; extracted CMYK values as feature data; and compared the performance of seven supervised algorithms: k-nearest neighbors (KNN), Gaussian Naive Bayes (GNB), global product classifier (GPC), RF, and artificial bee colony (ABC). The MLP algorithm achieved an accuracy of 0.82 and an F-measure of 0.81, outperforming the other algorithms. Subsequently, to enhance the MLP algorithm’s performance, they introduced GAN for data augmentation. This ultimately yielded the optimal algorithm, Aug-MLP (accuracy: 0.84, F-measure: 0.83), which demonstrated 100% accuracy in identifying antibiotics added to feed. The cause and mechanism of the fluorescence difference were identified using this array sensor.

Compared to traditional single-emission or dual-emission fluorescent probes, triple-emission fluorescent probes offer advantages, such as diverse color changes and reduced susceptibility to interference. Lu et al. [78] developed a portable device integrating a triple-emission fluorescent probe with ML-assisted smartphone technology, enabling quantitative and visual detection of TCs. The probe consists of blue-emitting carbon dots (BCDs) and red-emitting bovine serum albumin-protected copper nanoclusters (BSA-Cu NCs). The detection principle is based on the addition of different TCs, which induce shifts in the fluorescence peaks at 420 nm, 635 nm, and 520 nm, along with corresponding color changes, thereby enabling the detection of TCs. To achieve high-sensitivity, rapid, and convenient testing, they developed intelligent fluorescence analysis using YOLOv3 deep object detection. To extract and analyze color information, they compared six algorithms and selected different RGB channels for various TCs. Doxycycline hydrochloride (DC), Chlortetracycline Hydrochloride (CTC), and TC are suitable for the G channel, whereas OTC is suitable for the C/B channel. Finally, the least squares method was selected for fitting. To further demonstrate its application in real-world samples, they selected milk for testing TCs, achieving spiked recovery rates of 96.47–109.49%, highlighting the practicality of the sensor. Tan et al. [79] successfully synthesized a triple-emission ratio-based fluorescent probe and constructed a multimodal logic gate integrating fluorescence, colorimetric, and Ultraviolet detection channels. Specifically, they encapsulated CDs and Au NCs within ZIF-8 to synthesize a triple-emission ratio-based fluorescent probe (CD-Au NCs@ZIF-8). By integrating deep learning with smartphone tools, they achieved real-time, rapid identification of individual TCs. The detection principle involves the incorporation of TCs, which causes CD-Au NCs@ZIF-8 to exhibit altered fluorescence intensities at 440 nm and 640 nm and simultaneously generate a new fluorescence peak at 550 nm. Simultaneously, color changes occur. However, this only indicates the presence of TCs without enabling individual TC identification. Therefore, they developed a WeChat mini-program (96 Speckles) utilizing YOLOv5 and YOLOv8 algorithms for data processing and logic gate output. Ultimately, by incorporating different TCs, distinct variations in the ultraviolet–visible absorption spectrum peak of CD-Au NCs@ZIF-8 were achieved, thereby enabling the individual identification of the four TCs.

**Table 2:** Applications of ML in Antibiotic Detection.

Antibiotics Tested	Detection Mechanism	Probe	Probe Composition	ML	References
TC OTC DOX MTC	IFE	Dual-channel fluorescent sensor	QR-CDs, CPC-CDs	SVM, LDA	[49]
OFLX AMK PF NFC KNM DOX MTC TC SM	IFE, Static quenching, Electrostatic interactions	Dual-emission fluorescence/colorimetric sensor	CDs, CdTe quantum dots	TPTO, ERF	[76]
AMP CPFX KAN SMZ TET TMP	-	Nine-channel array	CNPs	Aug-MLP	[77]
DC CTC OTC TC	IFE, Sensitization mechanisms	Triple-emission fluorescent probes	BCDs, BSA-Cu NCs	YOLOv3, Least squares method	[78]
TTC OTC DC CTC	Sensitization mechanisms	Triple-emission fluorescent probes	CD-Au NCs	YOLOv5, YOLOv8	[79]

Table notes: TC, Tetracycline; OTC, oxytetracycline; DOX, doxycycline; MTC, minocycline; OFLX, ofloxacin; AMK, amikacin sulfate; PF, pefloxacin mesylate; NFC, norfloxacin; KNM, kanamycin sulfate; MTC, metacycline; SM, streptomycin; ERF, Extreme Random Forest; AMP, Ampicillin; CPFX, Ciprofloxacin; KAN, Kanamycin; SMZ, Sulfamethoxazole; TET, Tetracycline; TMP, Trimethoprim; DC, Doxycycline hydrochloride; CTC, Chlortetracycline Hydrochloride; TTC, Tetracycline.

#### 3.2.3. Application in the Detection of Certain Organic Compounds

**Figure 3:**
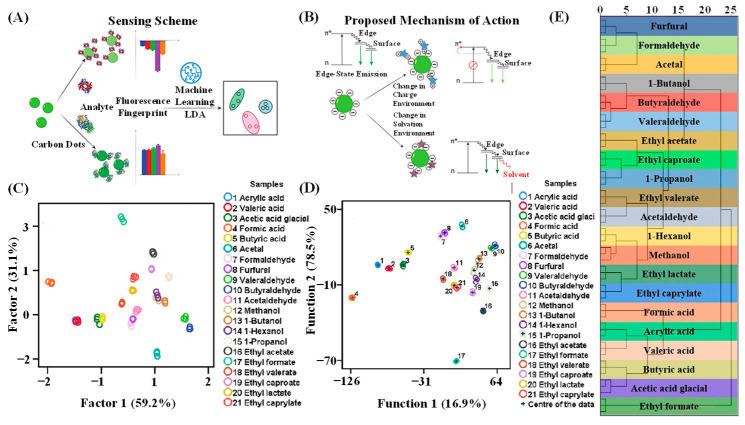
(**A**) Sensing events in CD array-based sensing. (**B**) Mechanism of CD fluorescence changes in the presence of proteins. Reprinted with permission from Ref. [40] Copyright 2019, American Chemical Society. Identification of 21 organic molecules: (**C**) PCA; (**D**) HCA; (**E**) LDA. Reprinted with permission from Ref. [81] Copyright 2023, Analytical methods.

ML technology can distinguish eight proteins with 100% accuracy. Pandit et al. [40] reported a biomolecular sensor based on a CD array for detecting proteins in buffers and human serum (Figure 3A). The detection principle relies on measurable fingerprint patterns generated by differential interactions with analytes, primarily driven by changes in the edge-state fluorescence of CDs, as illustrated in Figure 3B. At 460 nm, the eight proteins exhibited markedly different fluorescence changes in a buffer solution, enabling their identification based on these differences. To further determine the detection limit for proteins, they selected two representative proteins—hemoglobin (Hb) and β-galactosidase (β-Gal)—and observed distinct reaction patterns at varying concentrations in both PBS and human serum, exhibiting dose-dependent responses. ML algorithms outperformed LDA, with the final four ML algorithms achieving 100% accuracy. Ultimately, the detection limits for Hb and β-Gal in PBS were determined to be 25 nm, whereas those in human serum were found to be 50 nm.

ML can also assist in detecting bacteria. Soares et al. [80] developed an electrospun corn zein/curcumin carbon dot-based nanostructure electronic tongue for detecting *Staphylococcus aureus* in milk. This device performs comprehensive taste fingerprinting of liquid samples by combining sensor arrays with pattern recognition algorithms. During the detection process, they employed interactive document mapping (IDMAP) to reduce the dimensionality of capacitive spectra obtained from three sensor units (cCDOT, ZEI, and cCDOT/ZEI). This approach successfully distinguished pathogen samples at different concentrations from interfering samples. Subsequently, supervised learning algorithms (such as decision trees) were employed to construct a multidimensional calibration space (MCS), enabling automated classification and calibration. The study screened 57 frequency features using a decision tree model, generating an MCS for nine sample categories (including *Staphylococcus aureus* at varying concentrations and interferents) with an 86.1% classification accuracy. This model not only identified key distinguishing frequencies but also enhanced interpretability through rule visualization (ExMatrix). Based on this, they utilized the electronic tongue to distinguish between healthy cows, infected cows, and samples at different dilution levels, achieving a diagnostic accuracy of 80.1%. ML technology significantly enhanced the analytical capabilities of the carbon-dot sensor in complex environments, demonstrating its powerful potential for decision support throughout the process, from data dimensionality reduction to model calibration.

Deng et al. [81] employed a graphene quantum dots (GQDS) fluorescent sensor array. By utilizing a single GQDS sensing element combined with three different solvents (DMF: Dimethylformamide, DMSO: Dimethyl sulfoxide, EG: Ethylene glycol), they established a detection system capable of verifying the authenticity and quality of baijiu. They selected 21 organic compounds (acids, alcohols, aldehydes, and esters) present in baijiu at a concentration of 0.1 mM and analyzed them using PCA, HCA, and LDA. The first two principal components in the PCA algorithm account for a total of 90.3% of the information; however, the 21 organic compounds overlapped in the principal component plot, preventing the identification of all compounds (Figure 3C). Figure 3D,E demonstrate that HCA and LDA effectively distinguished all 21 organic compounds with 100% accuracy. Similarly, these methods can perfectly differentiate quaternary mixtures of methanol, acetaldehyde, butanoic acid ester, and ethyl acetate at various molar ratios.

### 3.3. Application of ML in Predicting the Performance of CDs

The preparation of CDs faces issues such as long development cycles, high costs, and unpredictability. ML can address these issues by building models that extract patterns from synthesis parameters and characterization data, thereby enabling rapid and accurate prediction of unknown properties. However, compared to density functional theory (DFT), the predictive performance of ML largely depends on the quality and breadth of the training data and lacks a physical interpretation. DFT, on the other hand, has advantages in terms of mechanistic interpretation and validation, making it suitable for fields such as mechanistic interpretation of materials and design validation. Nevertheless, the two approaches are not mutually exclusive. For example, Schleder et al. [82] discussed the relationship between ML and DF, showing that combining them can optimize computational workflows, thereby broadening the scope of materials research and advancing the field.

#### 3.3.1. Application in Predicting QY

In recent years, numerous studies have utilized ML to predict the yields of biochar and bio-oil from biomass, achieving significant progress. The high QY of CDs serves as a core metric for evaluating their luminescent performance. Chen et al. [41] pioneered the application of ML to predict the QY of CDs produced during biochar synthesis. First, they collected preparation parameters from 480 samples. First, based on the biochar production process, they identified 11 key parameters (cellulose, pyrolysis time, residence time, etc.). They collected preparation parameters for 480 samples under various combinations and split the dataset into a training set and a validation set in an 8:2 ratio. Subsequently, they evaluated six models, with the GBDT-R model demonstrating optimal QY prediction performance: R^2^ > 0.9, RMSE < 0.02 and MAPE < 3. Therefore, they employed the GBDT-R model for feature importance evaluation, identifying pyrolysis temperature, residence time, nitrogen content, and C/N ratio as the factors most significantly influencing QY. Furthermore, they simplified the parameters by selecting the top four features based on importance, with a relative error range of 0.00% to 4.60%. This model demonstrates good universality and provides a foundation for subsequent research on CD generation during biochar production.

**Figure 4:**
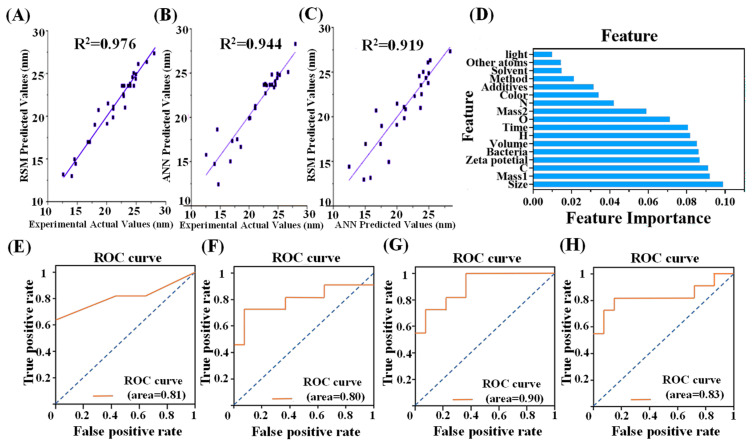
(**A**) RSM predictions of experimental values. (**B**) ANN predictions of experimental values. (**C**) Comparison of RSM and ANN predictions. Reprinted with permission from Ref. [84]. Copyright 2019, Multidisciplinary Digital Publishing Institute. (**D**) Gini importance of features in the raw data set and features after extraction. The ROC curves and AUCs for KNN (**E**), SVM (**F**), XGBoost (**G**), and RF (**H**), respectively. Reprinted with permission from Ref. [45]. Copyright 2024, International Journal of Nanomedicine.

Response surface methodology (RSM) is a statistical approach that integrates mathematical and statistical techniques based on polynomial equations and experimental data fitting. It predicts and optimizes multifactor systems by constructing response surface models [83]. Pudza et al. [84] employed a central composite design within RSM, selecting temperature, dosage, time, and solvent ratio as independent variables, with PLQY as the response value. When the optimal synthesis parameters were temperature 170 °C, dosage 0.1 g, time 100 min, and solvent volume 12 mL, the corresponding PLQY was 27.75%, with an RSM predicted value of 27.38% and R^2^ = 0.956. However, they acknowledged that while RSM effectively reduces experimental runs by examining interactions between factors, it has limitations in predicting nonlinear systems. ANN methods offer modeling capabilities for complex relationships. Therefore, they employed an ANN model from machine learning, pioneering the integration of the Levenberg–Marquardt backpropagation (LMBP) algorithm to construct a predictive model. This model incorporates temperature, dosage, time, and solvent ratio as inputs. After processing through hidden layers and optimizing the number of neurons, it outputs the PLQY, yielding a predicted value of 26.25% with R^2^ = 0.944. Figure 4A through Figure 4C demonstrate the reliability of this prediction method. The residual value between the RSM and ANN was ultimately found to be 0.0123, clearly indicating a high degree of consistency in the prediction results.

#### 3.3.2. Application at the Predicted Wavelength

ML integrates multiple synthetic parameters and can also achieve a precise prediction of CD emission colors and wavelengths. Senanayake et al. [85] first selected seven input features (precursor molar ratio, reaction temperature, reaction time, etc.), compiled a dataset using carbon nanodot synthesis data from the literature, and divided it into training and testing sets. They then employed an ANN as the core algorithm in ML to construct three machines (M1–M3), where M1 served as a regression model and M2 and M3 as classification models. Without considering reaction temperature and time, they found that the training mean error from the M1 machine was 16.3 nm, with a maximum standard deviation of 17.1 nm. Through various tests, it was observed that the M1 machine could accurately predict blue and green CDs but failed to accurately predict red CDs. Notably, M2 and M3 differed by leveraging the strong correlation between the “color” feature and emission wavelength. They fed the predicted color from the classification models into the regression model and applied a scoring algorithm to derive the predicted emission wavelength. Models M2 and M3 reduced the training mean absolute error to 9.8 ± 11.1 nm and 9.6 ± 10.5 nm, respectively, demonstrating significant effectiveness. Finally, after model construction and parameter tuning, the predicted color accuracy reached 94%, with a minimum average wavelength error of 25.8 nm. This achieved a precise prediction of the emission color and wavelength, thereby reducing experimental uncertainty.

Additionally, Yan et al. [86] applied ML to the preparation of phosphorescent CDs, achieving highly accurate predictions of CD emission wavelengths and Stokes shifts. First, they constructed a dataset comprising 210 datasets, with input variables including reaction time, sulfuric acid volume, water, and ethanol, and output performance metrics encompassing fluorescence and phosphorescence emission wavelengths and Stokes shifts. The dataset was then split into a training set and a validation set in an 8:2 ratio. Subsequently, they trained the machine learning models and evaluated their performance using R^2^, MAE, and RMSE. The XGBoost model demonstrated the best performance through comparison. Subsequently, the predicted values were compared with the experimental values. The R^2^ values for fluorescence and phosphorescence emission wavelengths were 0.95 and 0.94, respectively, while the R^2^ values for Stokes shifts were 0.89 and 0.94, respectively. This confirmed the high accuracy and robustness of the XGBoost model. Finally, to further validate the model’s performance, the authors varied the parameters to synthesize nine novel CDs. The results show that the predicted values agreed well with the experimental data, establishing a solid foundation for phosphorescent CDs in fields such as information encryption and bioimaging.

### 3.4. Application of ML in Studying the Mechanism of CDs

ML can uncover hidden patterns and causal relationships within complex, high-dimensional experimental data, thereby accelerating the revelation of the “structure-synthesis-property” relationship in CDs—a feat that is difficult to achieve through traditional trial-and-error methods. The performance of CDs (such as fluorescence and catalytic activity) is influenced by nonlinear, synergistic interactions among multiple factors: precursors, synthesis methods, and reaction conditions (temperature, time, pH, etc.). Traditional data analysis methods are unable to extract deep insights from such data. ML algorithms (such as RF and ANN) excel at handling high-dimensional, nonlinear datasets. They can simultaneously consider dozens or even hundreds of variables to identify the most critical features, thereby establishing predictive models between complex “synthesis conditions” and “final performance.” Extracting key patterns from complex data deepens our understanding of the mechanisms and directly guides the rational design and screening of new materials.

Salahinejad et al. [87] investigated the mechanism of fluorescence quenching in cysteine-functionalized carbon quantum dots (Cys-CQDs). They synthesized Cys-CQDs and measured their fluorescence intensity quenched by 25 heavy metal ions. By extracting the physicochemical parameters of the metal ions, they established correlations between these parameters and quenching sensitivity using quantitative structure–property relationship (QSPR) models. Descriptor selection is fundamental to QSPR modeling. To extract more information, they employed stepwise regression, genetic algorithm (GA), and enhanced replacement method (ERM) techniques from machine learning. Simultaneously, they utilized multiple linear regression (MLR) and SVM to establish the QSPR model. The ratio of the luminescence peak area $I_{0}$ (without metal ions added to the Cys-CQD solution) to $I_{Q}$ (after addition) was used as the dependent variable in the QSPR model, and the SVM model outperformed the MLR model. ERM then extracts meaningful descriptions from the set of descriptors through iterative substitution and combinatorial optimization. Ultimately, ERM provides the covalent index (CovIn), atomic environment number (AEN), and number of valence electrons (NVE) as the optimal subset of descriptors. Equation (5), derived from the MLR, indicates that the key parameters influencing heavy metal ion-induced fluorescence quenching of Cys-CQDs, in order of importance, are CovIn, AEN, and NVE. This elucidates the structure–activity relationship governing CQD fluorescence quenching and provides theoretical guidance for designing highly selective metal fluorescent probes.

(5) $$\frac{I_{0}}{I_{Q}}=\left(2.643\pm 0.231\right)CovIn+\left(0.275\pm 0.051\right)AEN-\left(0.235\pm 0.027\right)NVE-\left(5.115\pm 0.863\right)$$

Furthermore, since the discovery of solid-state phosphorescence properties at room temperature in carbon-based quantum dot materials, such as carbon dots and graphene quantum dots, elucidating their phosphorescence mechanisms has remained a significant challenge in studying structure–property relationships. Li et al. [88] introduced ML to establish a quantitative structure–property relationship to explore the phosphorescence mechanism of GQDs. They introduced structural variability (Ω), porosity (V), and disorder (S). The equation is

(6) S=2lnΩ+V.

Subsequently, they analyzed the structural disorder using ML and quantified the structural features. Simultaneously, they discovered that S is inversely correlated with the phosphorescence lifetime. Finally, they demonstrated that S can characterize the oscillator strength, thereby enabling the precise regulation of phosphorescence properties based on this finding.

CDs have emerged as alternative antimicrobial agents because of their unique physicochemical properties. Bian et al. [45] employed ML tools to investigate the relationship between the physicochemical characteristics of CDs and their antimicrobial capabilities, aiming to elucidate the underlying mechanisms. First, they constructed a dataset comprising 121 CD samples, which were processed to retain 17 features. The minimum inhibitory concentration (MIC) was used to determine antimicrobial activity. Next, feature importance was calculated. As shown in Figure 4D, the size of CDs, zeta potential, and bacterial species exerted the greatest influence on antimicrobial performance. This finding helps explain the relationship between physicochemical properties and antimicrobial efficacy. Additionally, they employed four ML classification algorithms—KNN, RF, XGBoost, and SVM—with MIC as the output metric, and evaluated the models based on the area under the ROC curve and accuracy (Figure 4E–H). The XGBoost model achieved an accuracy of 78.3% and an AUC of 0.90, indicating that it is the optimal model. Finally, they applied this model to predict ε-poly-L-lysine CDs (PL-CDs), thereby accelerating the design of highly effective antimicrobial CDs.

## 4. Conclusions and Perspective

ML has evolved from an emerging computational paradigm into a disruptive force driving progress across multiple disciplines. As a powerful data analysis technique, ML has deeply penetrated CD research, demonstrating immense potential. This study reviews recent ML applications in CDs, focusing on ML-optimized CD synthesis, sensor detection, predictive performance, and mechanistic studies. Generally, supervised learning is widely used in carbon dot research, but unsupervised learning also has unique value in specific contexts [14]. By establishing mapping models between synthesis parameters (such as precursor type, reaction temperature, time, and solvent) and target properties (QY and emission wavelength), ML effectively predicts optimal synthesis conditions, thereby accelerating the preparation of high-performance CDs. Concurrently, ML algorithms (such as support vector machines and convolutional neural networks) have been employed to process and analyze complex data generated by CD sensors (such as fluorescence spectra and images), thereby enabling high-precision, rapid identification and classification of multiple analytes and significantly enhancing the intelligence of detection. The reliability of ML depends on rigorous data and model optimization and therefore has inherent limitations. The current application of ML in CD research still faces several key challenges that limit its further development and broader application.

First, current research is generally constrained by small sample datasets (typically only a few hundred datasets), which severely limits the generalization capability and reliability of models. Future efforts should focus on expanding datasets at scale through high-throughput experiments and automated data collection. The field of CDs lacks a unified, open-source standard database. There is an urgent need to establish a standardized database containing precise synthesis conditions, detailed microstructural characterization (such as surface functional groups and particle size distribution), and multifunctional performance data. Promoting data format interoperability is also essential to facilitate data sharing. Differences in the size, shape, and anchoring methods of functional groups on CDs can easily lead to biases in model interpretation, feature redundancy that reduces accuracy, and confusion regarding the mechanisms of action of these functional groups. In practical applications, ML models cannot be applied directly; instead, feature engineering should be optimized by taking into account the surface structure of the CDs and the characteristics of their functional groups. Furthermore, current ML models are largely “black boxes,” lacking explanations for the intrinsic physicochemical mechanisms underlying the structure–property relationships of CDs. Future efforts should closely integrate theoretical calculations, incorporate structural parameters as model inputs to enhance interpretability, and develop multi-objective optimization algorithms to simultaneously regulate multiple performance metrics, such as emission wavelength, quantum yield, and lifetime. Finally, in practical applications, models should be further optimized to improve detection stability and reliability. Moreover, this approach can be extended to other applications, building a universal visualization sensing platform. For instance, in ion detection, the detection range can be expanded to cover more ions. Furthermore, applying this method to real-world environmental samples will enable the continuous optimization of detection techniques for increasingly complex environments. In the context of rapid AI advancements, we believe that machine learning—as a core branch—will infuse nanomaterials science with new intelligent vitality, achieving breakthrough applications in the field of carbon nanotubes.

## Data Availability

No new data were created or analyzed in this study.
